# Small bowel metastasis during organ preservation in patients with esophageal squamous cell carcinoma: case report and literature review

**DOI:** 10.3389/fonc.2025.1560011

**Published:** 2025-07-24

**Authors:** Qinxing Cao, Hejiang Lu, Liangbo Niu, Xiaofang Zhao, Li Yan, Yisha Liu, Minghui Pang

**Affiliations:** ^1^ Department of Gastrointestinal Surgery, The Affiliated Hospital, Southwest Medical University, Luzhou, China; ^2^ Department of Geriatric General Surgery, Sichuan Provincial People’s Hospital, School of Medicine, University of Electronic Science and Technology of China, Chengdu, China; ^3^ Department of Radiology, The First Affiliated Hospital of Chongqing Medical University, Chongqing, China

**Keywords:** esophageal squamous cell carcinoma, small bowel metastasis, clinical complete response, watch-and-wait, organ-preserving treatment

## Abstract

**Background:**

For patients with locally advanced esophageal squamous cell carcinoma (ESCC) who achieve a clinical complete response (cCR) after comprehensive treatment, organ preservation and observation-waiting strategies provide a more conservative treatment option that enhances the patient’s quality of life. The high cCR rate of chemoradiotherapy combined with immunotherapy is driving the shift in ESCC treatment from traditional radical surgery to organ preservation.

**Case summary:**

We report a case of a 57-year-old male patient diagnosed with esophageal ESCC who underwent combined radiotherapy, chemotherapy, and immunotherapy. After treatment, the patient achieved a cCR, resulting in 15 months of progression-free survival. At this stage, the primary lesion showed no signs of local regrowth or recurrence; however, unexpected metastasis to the small intestine occurred, leading to bowel obstruction. The metastasis at this occult site was not detected by sensitive monitoring methods, and the side effects of the immunotherapy drugs further complicated the diagnosis and differential diagnosis. The tumor metastasis at the unexpected site was not identified early, but following rescue surgery, the patient survived for an additional 6 months.

**Conclusions:**

Organ-preserving surgery for esophageal cancer significantly improves patients’ short-term quality of life. However, owing to incomplete monitoring measures, a cautious approach should be maintained when implementing organ-preserving surgery at this stage. For patients undergoing organ-preserving surgery, continuous active monitoring is essential. Timely intervention should be provided when clinical symptoms arise, and personalized treatment plans should be developed, while remaining vigilant for metastasis at unexpected sites.

## Introduction

Esophageal squamous cell carcinoma (ESCC) is one of the most common malignant tumors of the human esophagus (1). Platinum-based chemotherapy is the conventional treatment modality, though its effectiveness is limited (2). Immune checkpoint inhibitors (ICIs) as a new form of immunotherapy have shown excellent response rates in certain intractable cancers and are widely used in clinical practice, gradually changing the treatment paradigm. However, selecting an appropriate treatment strategy in clinical practice remains a significant challenge. Despite the rapid advancements in precision medicine technologies, the prognosis for ESCC remains poor. Therefore, considering how to leverage these new technologies to provide doctors with appropriate guidance strategies is something worth pondering.

Radical surgery remains the primary treatment for patients with advanced esophageal cancer. However, esophageal surgery is complex, causes significant trauma, requires a long recovery period, and leads to poor quality of life—issues that cannot be overlooked. In recent years, neoadjuvant chemoradiotherapy combined with immunotherapy has shown promise in treating esophageal cancer, offering higher pathological complete response (pCR) rates and potential for organ preservation. The NEOCRTEC 5010 (3) study confirmed that neoadjuvant chemoradiotherapy significantly improved pCR rates and extended both median overall survival (OS) and progression-free survival (PFS) compared to surgery alone in patients with ESCC. The FFCD 9102 (4) study found that for patients with ESCC who responded well to chemoradiotherapy, surgery did not provide additional survival benefits over continued chemoradiotherapy. The CheckMate-577 (5)study showed that postoperative adjuvant immunotherapy significantly prolonged PFS (22.4 months vs. 11.0 months). The PALACE-1 (6)study reported a pCR rate of 55.6% with chemoradiotherapy combined with immunotherapy. The KEYNOTE-590 (7) study indicated that adding immunotherapy improved both OS and PFS in patients with advanced esophageal cancer. Therefore, it remains controversial whether patients with esophageal cancer who achieve a clinical complete response (cCR) after neoadjuvant therapy or systemic induction therapy still need surgery, and large-scale clinical studies to confirm this are currently lacking.

We report a case of a patient with mid-thoracic ESCC who achieved a cCR in both the primary tumor and lymph node metastases following systemic induction therapy. The patient chose a watch-and-wait strategy. During maintenance immunotherapy, the patient developed metastasis to an unexpected site (small intestine), while the primary tumor remained in complete clinical response. As chemoradiotherapy combined with immunotherapy progresses, more patients are choosing an observation-waiting strategy to improve their quality of life.

In the near future, metastasis to unexpected sites during the watch-and-wait period in patients with esophageal cancer is likely to become more common. This case underscores the need for updated monitoring methods and follow-up strategies for patients with esophageal cancer after organ-preserving treatment, highlighting the importance of developing comprehensive evaluation and monitoring approaches for unexpected site metastasis. In clinical practice, special attention should be given to distinguishing metastases from adverse events caused by chemoradiotherapy or immunotherapy.

## Case presentation

**Figure 1:**
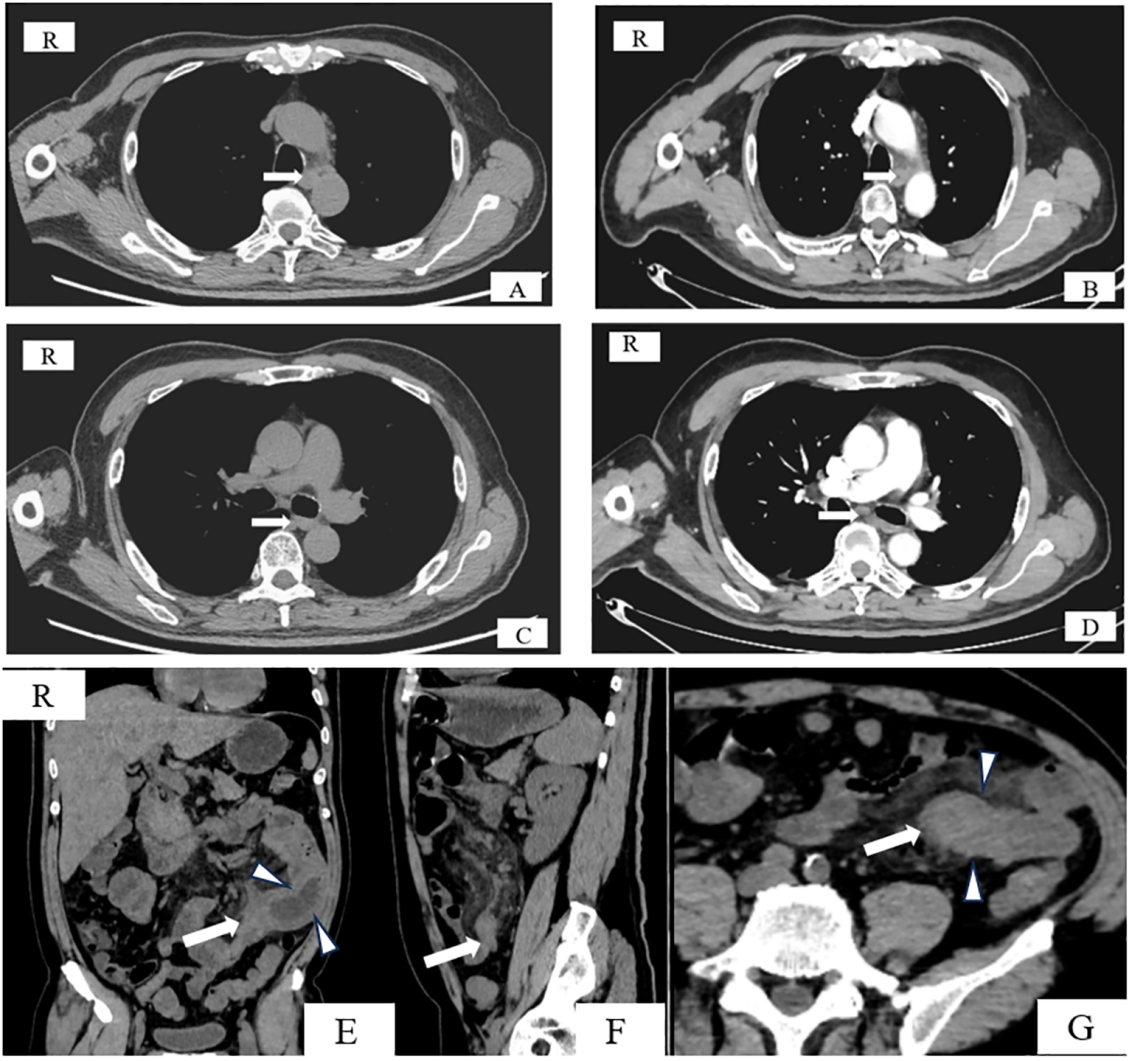
CT images. **(A, B)** Axial CT images of the esophageal primary tumor, 1 month preoperatively **(A)** and preoperatively **(B)**, showing no significant changes. **(C, D)** Axial CT images of lymph nodes below the tracheal carina, 1 month preoperatively **(C)** and preoperatively **(D)**, showing no significant changes. **(E–G)** Coronal, sagittal, and axial CT images demonstrating uneven thickening of the intestinal wall (short arrow), luminal narrowing, and occlusion, with an obstructive point visible in the distal portion (long arrow).

On 15 August 2023, a 56-year-old male patient was admitted to the hospital with a chief complaint of “abdominal pain, abdominal distension, accompanied by vomiting, and no passage of gas or stool for 4 days.” It is noteworthy that he had been admitted on 23 March 2022, due to difficulty swallowing and was diagnosed with advanced ESCC of the mid-thoracic esophagus. Enhanced chest and abdominal computed tomography (CT) revealed regular thickening of the mid-thoracic esophagus with a local mass, measuring approximately 2.3 cm × 2.8 cm, suggesting esophageal cancer with multiple lymph node metastases, with no obvious abnormalities in the abdomen. Positron emission tomography–computed tomography (PET-CT) examination (as shown in **Figure 1**) indicated esophageal cancer with lymph node metastases in the bilateral supraclavicular and left upper tracheal regions, with no obvious abnormalities in other regions, including the abdomen. The patient’s personal history, surgical history, and family history were unremarkable.

According to American Joint Committee on Cancer/Union for International Cancer Control Tumor-Node-Metastasis (AJCC/UICC-TNM) staging (8th edition), the preoperative diagnosis is clinical stage IVB ESCC (cT3, cN1, cM1). Following a multidisciplinary assessment, the patient is currently not a candidate for surgical treatment. Therefore, the therapeutic objective is disease control rather than downstaging for surgery.

A personalized systemic induction therapy plan was developed based on the results of comprehensive tumor landscape gene testing, circulating tumor DNA (ctDNA) testing, and programmed death-ligand 1 (PD-L1) testing (as shown in **Tables 1**-**3**). The first systemic induction therapy began on 20 March 2022, and targeted ctDNA tracking was conducted during this period (as shown in **Table 2**). The specific systemic induction therapy regimen includes pembrolizumab 200 mg on day 1 intravenously every 3 weeks, albumin-bound paclitaxel 0.3 g on day 1 intravenously every 3 weeks, and carboplatin 300 mg on day 1 intravenously every 3 weeks.

**Table 1:** Summary of comprehensive genomic profiling results in solid tumors.

Item	Test Result Overview	Detailed Test Results	Clinical significance in treatment, prognosis, and diagnosis
Genomic mutation testing			
Tier 1 mutations	0		Strong clinical significance (Grade A/B evidence)
Tier 2 mutations	2	*PIK3CA*, *TP53*	Potential clinical significance (Grade C/D/E evidence)
Tier 3 mutations	12	*CCND1*, *CDKN2A*, *FBXW7*, *FGF19*	Significance location or unreported
Immunotherapy predictive assessment			
MSI		MSS	Indicative of prognosis and assist in treatment planning
TMB		4.87 mutations/Mb (Intermediate)	Evaluate the effectiveness of immune checkpoint
Potential therapeutic c	Targets cancer-specific	Positive gene (0) Negative gene (0) Hyperprogression genes (4)	Assess the efficacy of immune drugs targeting
	Other cancer-related positive genes	Positive gene (2) Negative gene (4) Hyperprogression genes (0)	—
HLA-I	HLA-I (A/B/C)	Partial homozygous	Predict the effectiveness of immunotherapy
DDR-related gene mutations	*TP53*		PARP/Platinum-based/immune checkpoint
Mutation testing results	1	TP53ρ.R273H	Evaluate the effectiveness of PARP inhibitors
Tumor genetic susceptibility			
Genetic susceptibility screening	0	—	—

**Table 2:** Summary of ctDNA testing results.

Sample type	*p*-Value^3^	ctDNA content^2^ (hGE/mL)	MRD interpretation
Plasma (before 1 cycle of treatment)	<1.00*10^-4^	2.23	Positive ^1^
Plasma (after 3 cycles of treatment)	<1.70*10^-4^	1.33	Positive
Plasma (after 4 cycles of treatment)	<1.00*10^-4^	6.14	Positive

^1^ MRD positive means the presence of circulating tumor DNA in the blood, indicating a high risk of recurrence and poor prognosis when positive. ^2^ ctDNA content refers to the average amount of tumor molecules in each milliliter of plasma and is related to tumor burden. ^3^ *p*-value is used to measure the significance of ctDNA positivity in plasma. A *p*-value ≤ 0.01 is interpreted as positive.

**Table 3:** Initial pathology and PD-L1 testing at the first visit.

Item	Pathology 25 cm from incisors	PD-L1 testing 25 cm from incisors
Diagnosis	Esophageal squamous cell carcinoma	CPS (combined positive score) = 1
Testing method	H&E staining	PD-L1 clone antibody (clone number 22C3)
Control conditions	——	Positive control: positive; negative control: negative

After four cycles of systemic induction therapy treatment, PET-CT assessment showed stability of the primary lesion. On 4 July 2022, the treatment approach was switched to systemic induction therapy chemoimmunotherapy and concurrent radiotherapy, with the specific regimen consisting of pembrolizumab 200 mg on day 1 intravenously every 3 weeks, albumin-bound paclitaxel 0.3 g on day 1 intravenously every 3 weeks, and carboplatin 300 mg on day 1 intravenously every 3 weeks, and primary lesion plus bilateral supraclavicular lymph node metastatic foci received local radiation therapy, with a single dose of 1.8 Gy. Following six cycles of chemoimmunotherapy, 28 sessions of radiotherapy, and five cycles of pembrolizumab maintenance therapy, a follow-up chest and abdominal CT scan and gastric endoscopy on 29 November 2022 indicated near-complete clinical response (near-cCR) of the primary lesion. Therefore, pembrolizumab maintenance therapy was continued.

### First episode of intestinal obstruction

On 17 July 2023, the patient was admitted for “abdominal pain with cessation of bowel movements.” Based on the medical history, physical examination, and auxiliary investigations, an incomplete small bowel obstruction was diagnosed; abdominal CT favored an inflammatory etiology. In the hospital, the patient received antibiotics, nutritional support, gastrointestinal decompression, and enemas. After treatment, abdominal pain resolved, flatus and defecation resumed, and a liquid diet was tolerated without discomfort; thus, the patient was discharged.

### Second episode of small bowel obstruction

On 15 August 2023, the patient was readmitted for “abdominal pain accompanied by cessation of bowel movements.” Vital signs were as follows: temperature 36.4°C, heart rate 110 beats/min, respiratory rate 18 breaths/min, and blood pressure 91/69 mmHg (1 mmHg = 0.133 kPa). Bilateral breath sounds were clear, with no wheezes or moist rales, and the heart rhythm was regular. The abdomen was mildly distended without visible peristaltic waves; no masses were palpable. There was upper-abdominal tenderness without muscle guarding or rebound tenderness. Percussion revealed tympany throughout, and bowel sounds were about six per minute. No abnormal cervical lymph nodes were detected.

Tumor markers were as follows: CEA <1.73 ng/mL (normal range <5.0 ng/mL), CA199 2.81 U/L (normal range <43 U/L), and NSE 20.20 ng/mL (normal range ≤16.30 ng/mL). An enhanced CT scan on 15 August 2023 (**Figures 1E–G**) indicated uneven thickening of the intestinal wall in the left abdomen causing luminal narrowing, with consideration of a tumor or inflammatory lesion causing proximal intestinal obstruction. Combining clinical presentation with auxiliary examination results, small bowel obstruction was suspected. Comparison with CT scans from 7 February 2023 (external examination), 20 July 2023, and 15 August 2023 (**Figures 1A–D**) showed no significant changes in the esophageal lesion, indicating treatment-related alterations and the efficacy assessment remained complete remission.

### Diagnosis

Based on medical history, physical examination, and ancillary investigations, the following diagnoses were established: small intestinal mass with obstruction, esophageal carcinoma (ycT0N0M0), post-chemoradiotherapy status for esophageal carcinoma, and post-immunotherapy status for esophageal carcinoma.

#### Differential diagnoses

##### Primary small intestinal tumor

Mainly gastrointestinal stromal tumors (GISTs) and adenocarcinomas; small intestinal GISTs have a comparatively higher malignant potential.

##### Chronic intestinal pseudo-obstruction

Clinically mimics mechanical obstruction, but no true obstructive lesion is present; thus, it must be differentiated from genuine obstruction.

#### Treatment

Considering the patient’s medical history, physical examination, and relevant auxiliary examinations, it was determined that there was an indication for surgery. On 21 August 2023, the patient underwent an emergency exploratory laparotomy.

**Figure 2:**
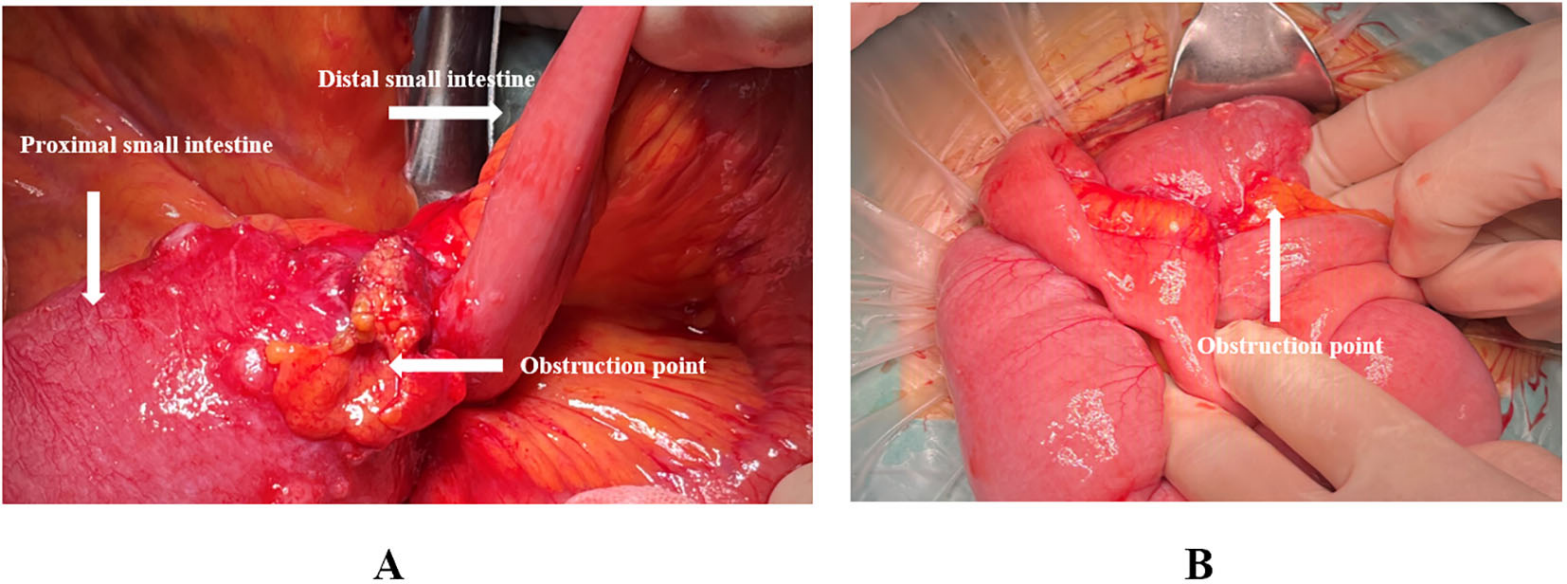
**(A, B)** Intraoperative images showing the location of the obstruction point, located 80 cm from the Treitz ligament.

Exploration revealed no visible nodules on the falciform ligament, lesser omentum, right and left upper abdomen, diaphragmatic peritoneum, parietal peritoneum, bilateral iliac fossae, or pelvic floor peritoneum. A 2-cm intraluminal tumor was noted 40 cm distal to the Treitz ligament, and another 5-cm lesion was identified 80 cm distal to the Treitz ligament. Both tumors had penetrated the serosa and were firm in texture. The 5-cm lesion (80 cm from Treitz) was densely adherent to the transverse-colonic omental fat and distal ileum, creating a constrictive ring with marked proximal dilatation and fluid accumulation—presumed to be the mechanical cause of obstruction (**Figure 2**). Multiple scattered, firm, white nodules (≈0.1–0.3 cm) were seen in the greater omentum, small intestinal, and colonic mesentery; intraoperative frozen sections confirmed them as metastatic small bowel carcinoma. Taken together, the findings are consistent with peritoneal carcinomatosis, and the calculated Peritoneal Cancer Index (PCI) is 8.

As the procedure was performed on an emergency basis, the patient had a preoperative ECOG performance status of 3 and poor nutritional reserve, rendering radical cytoreductive surgery high-risk. Accordingly, based on the intraoperative findings, we undertook segmental small bowel resection with anastomosis, omentectomy, peritoneal lesion excision with biopsy, and intraoperative intraperitoneal perfusion of raltitrexed.

**Figure 3:**
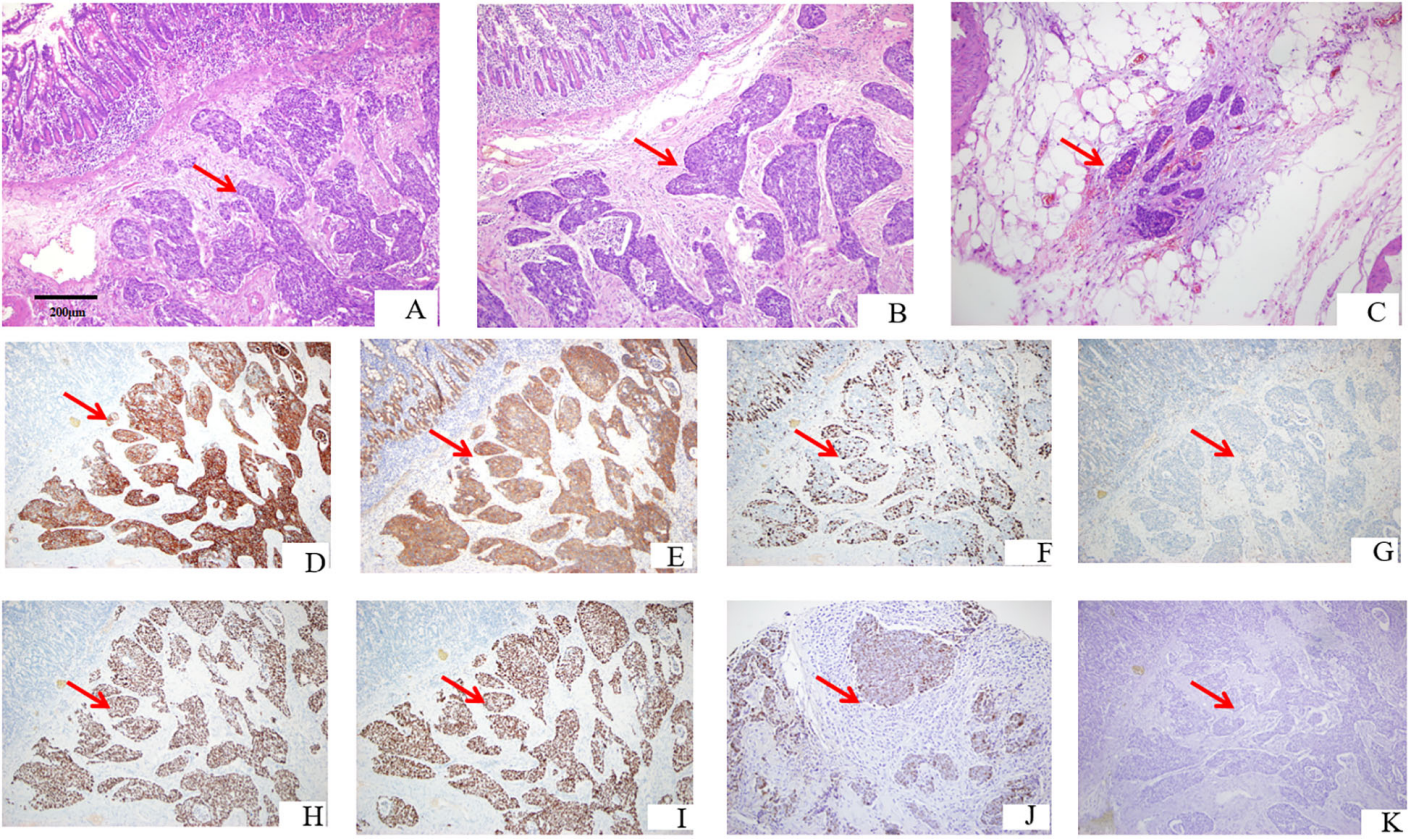
Pathological and immunohistochemical results. **(A)** Intestinal ① shows tumor cell infiltration HE × 100. **(B)** Intestinal ② shows tumor cell infiltration HE × 100. **(C)** Intestinal ① shows the presence of cancer nodules HE × 100. **(D)** CK5/6 positive immunohistochemical staining × 100. **(E)** EGFR-positive immunohistochemical staining × 100. **(F)** Ki-67-positive rate 30% immunohistochemical staining × 100. **(G)** P16-negative immunohistochemical staining × 100. **(H)** P30-positive immunohistochemical staining × 100. **(I)** P63-positive immunohistochemical staining × 100. **(J)** EBER *in situ* hybridization-positive external control immunohistochemical staining × 100. **(K)** EBER *in situ* hybridization-negative immunohistochemical staining × 100.

**Figure 4:**
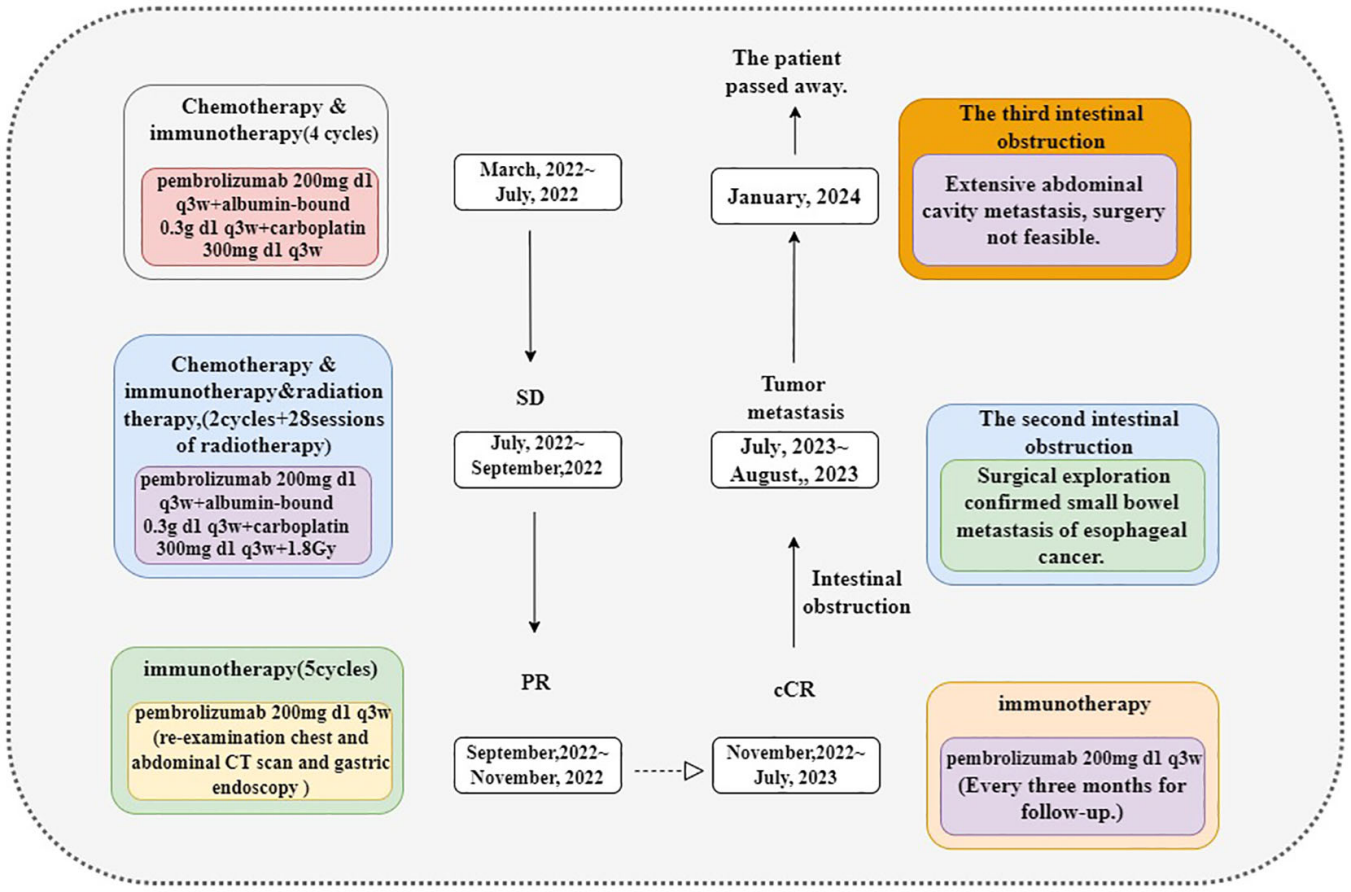
Here is the timeline of our case treatment.

Postoperatively, the diagnosis of secondary ESCC was confirmed by combining H&E staining (**Figures 3A–C**) and immunohistochemistry, accompanied by a vascular system with numerous tumor emboli and the formation of cancer nodules in the mesentery tissue. Considering the patient’s medical history, it is believed that this tumor originated from esophageal cancer. Immunohistochemistry (**Figures 3D–K**): CK5/6(+); EGFR(+); Ki-67(20%+); P16(−); P40(+); P63(+); ISH: EBER1/2(−). Based on the patient’s condition, solid tumor genetic testing and MSI high-throughput sequencing were performed postoperatively, postoperative genetic testing results are shown in **Table 4**, and the next treatment plan was formulated based on the pathological and genetic testing results. **Figure 4** briefly depicts the patient’s full disease course and key treatment milestones.

**Table 4:** Postoperative 29-gene tumor profiling and MSI testing.

Project	Test results	Significance	Source of evidence
*TP 53*	*TP53ρ.R273H*	Adavosertib sensitivity	Clinical study
*UGT1A1*	*(TA)6/(TA)6*	Low risk of diarrhea and neutropenia with irinotecan use	
MSI	MSS	/	/

## Discussion

Although surgery remains the standard treatment for resectable locally advanced esophageal cancer, organ preservation has gained increasing attention with advances in drug therapies and radiotherapy techniques. However, controversy remains regarding the benefits of organ preservation for patients.

In the CROSS (8) and NEOCRTEC 5010 (3)studies, the pCRrates in patients with ESCC after concurrent chemoradiotherapy were 49% and 43%, respectively, providing a theoretical basis for organ preservation. The FFCD 9901 (4) trial, which explored organ preservation, also showed that adding surgery for patients with ESCC who achieved pCR did not provide additional survival benefits. The 2023 ESMO-released SANO (9) study further addressed this issue, indicating no significant differences in OS and disease-free survival (DFS) between immediate surgery and active monitoring after neoadjuvant chemoradiotherapy. Moreover, organ preservation significantly improved patients’ short-term quality of life. Immunotherapy has shown promising results in advanced esophageal cancer and perioperative treatment, with the PALACE-1 study (6) suggesting that adding immunotherapy to concurrent chemoradiotherapy further enhances the pCR rate (56%). Therefore, for patients achieving cCR after neoadjuvant therapy, the strategy of organ preservation with close follow-up and monitoring is theoretically feasible.

The development of neoadjuvant chemoradiotherapy combined with immunotherapy has enhanced the feasibility and safety of organ preservation in advanced esophageal cancer, prompting more patients to choose an observation-waiting strategy after achieving cCR (10, 11). However, not all patients benefit from organ preservation; some even experience hyperprogression after immunotherapy. Among those who benefit from organ preservation, recurrence and metastasis still occur (12, 13). The biggest challenge in organ preservation is the accuracy of detecting and assessing esophageal cancer lesions, as existing detection methods have limited sensitivity in identifying residual disease. The preSANO (14) and preSINO (15) studies explored the effectiveness of combined assessments using imaging, endoscopy, and biopsy. However, most of these studies focus on evaluating the primary tumor and common metastatic sites (lymph nodes, lungs, liver, bones, adrenal glands, and brain), with few addressing unexpected metastatic sites.

This case presents an esophageal cancer metastasis to an unexpected site, with “intestinal obstruction” as the initial symptom, posing significant challenges in clinical diagnosis and treatment strategies. One study showed that unexpected metastasis can occur at any stage of esophageal cancer, with metastatic lesions (16) primarily spreading to five anatomical sites: head and neck (42%), chest (17%), abdomen and pelvis (25%), limbs (9%), and skin and muscle metastases (7%), with a median OS of approximately 10.2 months. These unexpected metastases may be closely related to the unique anatomy of the esophagus, its special vascular system, and lymphatic skip metastasis (retrograde and bidirectional) (17–20).

In this case, multiple firm, white nodules were identified intraoperatively on the small intestinal mesentery and serosal surfaces. Pathological examination confirmed these to be metastatic carcinoma, consistent with peritoneal carcinomatosis. Although such metastases are relatively uncommon in ESCC, they are receiving increasing clinical attention in the era of immunotherapy-driven survival prolongation. Current literature has largely focused on locally advanced ESCC, with limited discussion on the organ-preservation needs and metastatic risks in patients with advanced disease who achieve cCR after multimodal therapy. As more patients with advanced or locally advanced ESCC achieve cCR, this case highlights the importance of vigilance for unexpected metastatic spread and the need to refine existing surveillance strategies.

To evaluate the extent of peritoneal involvement, we applied the PCI, a widely used tool in tumor staging and surgical planning (21). The PCI score in this case was 8, indicating moderate peritoneal disease. The Peritoneal Surface Disease Severity Score (PSDSS) was calculated as 9, suggesting a poor prognosis (22). Given the patient’s overall condition, cytoreductive surgery with hyperthermic intraperitoneal chemotherapy (CRS+HIPEC) was not feasible (23). This underscores the occult nature of metastases in uncommon anatomical sites and the challenge of timely detection, contributing to poorer outcomes.

Given the above, patients undergoing organ-preserving treatment require close monitoring during and after treatment. However, assessing these patients remains challenging. The preSANO (14)study suggests that endoscopic ultrasound, deep biopsy, and lymph node fine needle aspiration are effective for evaluating local residual disease in achieving cCR. However, these methods have high sampling requirements and are prone to false negatives. Regular PET-CT scans are recommended after treatment to monitor lymph node and distant metastasis, though the optimal monitoring interval remains undefined. Building on preSANO, the preSINO (15) study incorporated ctDNA testing, showing that ctDNA-positive patients have significantly higher rates of distant recurrence than ctDNA-negative patients, thereby reducing the false-negative rate. However, current research primarily focuses on monitoring the primary tumor, with limited studies on unexpected metastatic sites. More sensitive molecular imaging, liquid biopsy, and other precise non-invasive evaluation tools are still needed. Considering the limitations of conventional imaging in the post-immunotherapy setting, emerging artificial intelligence-based methods may facilitate earlier detection and risk prediction of peritoneal carcinomatosis through multimodal integration. Recent studies in gastric (24) and colorectal cancers (25) have shown promise in improving the accuracy of peritoneal metastasis prediction. Integrating various data sources—such as liquid biopsy, ctDNA dynamics, and molecular imaging (e.g., ^68^Ga-labeled agents or advanced MRI)—with machine learning models may significantly enhance follow-up protocols and optimize organ-preservation strategies in advanced ESCC.

The small intestine has a highly efficient local immune response, characterized by a large number of lymphocytes within the submucosa and intestinal epithelium, as well as the secretion of immunoglobulins such as IgA, which play a crucial role in reducing tumor incidence (26). Consequently, small bowel metastasis occurs infrequently, accounting for less than 2% of cases (27). In this instance, the patient developed small bowel obstruction during maintenance therapy with a PD-L1 inhibitor. The administration of PD-L1 inhibitors can induce immune-related adverse events (IRAEs), including colitis, enteritis, paralytic ileus, and chronic subacute intestinal obstruction. IRAEs are relatively common in patients treated with ICIs, with an incidence rate exceeding 60% (28). Furthermore, studies have demonstrated that anti-PD-L1 treatment may result in delayed adverse events, which can persist even after treatment has been discontinued (28–30).

In this case, the patient’s local tumor remained in remission, and the occurrence of metastasis at an unexpected site during the observation period represents an extremely rare situation. Initially, secondary small bowel tumors were not identified upon the patient’s admission due to the stability of the primary lesion and the atypical clinical and radiological presentations of early metastasis, which led to the consideration of IRAEs. Various abdominal inflammatory conditions produced artifacts that complicated the preoperative abdominal evaluation, posing significant challenges in the timely detection of small bowel metastasis. When managing esophageal cancer, it is crucial to emphasize the importance of imaging screening, especially for patients with gastrointestinal symptoms and a relevant medical history. Therefore, patients with a history of esophageal cancer who develop acute abdominal pain during chemotherapy or immunotherapy should undergo a comprehensive evaluation of the small intestine and intra-abdominal conditions to avoid overlooking occult metastasis and delaying necessary treatment. Additionally, after obtaining informed consent, the consideration of laparoscopic exploration or surgery may be helpful in diagnosing cases of acute abdominal pain.

Most patients undergoing organ-preserving surgery for esophageal cancer may experience local recurrence. For these patients, continuous active monitoring enables safer and more efficient subsequent surgeries in the event of recurrence (14, 15). However, for patients with metastasis to unexpected sites, even with continuous active monitoring, it can still be challenging to detect occult metastasis at these sites in a timely manner, particularly when intra-abdominal metastasis occurs, which may result in a worse prognosis compared to patients with local recurrence. Despite the poorer prognosis for these patients, our patient survived for 6 months after surgical intervention. We believe that enhanced monitoring for unexpected metastatic sites in esophageal cancer is critical. For patients presenting with clinical symptoms, timely surgical intervention and personalized adjuvant therapy can play a pivotal role in improving prognosis.

## Conclusion

Esophageal cancer surgery is highly invasive, resulting in a significant decline in postoperative quality of life. Organ-preserving surgery can better maintain eating and swallowing functions, thereby improving quality of life. Current detection methods have limited sensitivity for small residual lesions, and research on monitoring unexpected metastatic sites remains insufficient, potentially necessitating more sensitive technologies such as molecular imaging and liquid biopsy. At this stage, the observation and waiting strategy should be approached with caution, and patients with a maintained cCR of the primary lesion should remain vigilant for metastasis at unexpected sites. In the near future, through continuous active monitoring, early identification of recurrence and metastasis, timely surgical intervention, and personalized adjuvant treatment strategies, patient prognosis and quality of life can be significantly improved.

## Data Availability

The original contributions presented in the study are included in the article/**Supplementary Material**. Further inquiries can be directed to the corresponding author.
