# Supplementary material for: Small bowel metastasis during organ preservation in patients with esophageal squamous cell carcinoma: case report and literature review
Source: Front Oncol. 2025 Jul 24;15:1560011. doi: 10.3389/fonc.2025.1560011 (PMC12328174; doi:10.3389/fonc.2025.1560011)
Supplement: Supplementary file 1 [file DataSheet1.docx]

**Additional file-Figure**


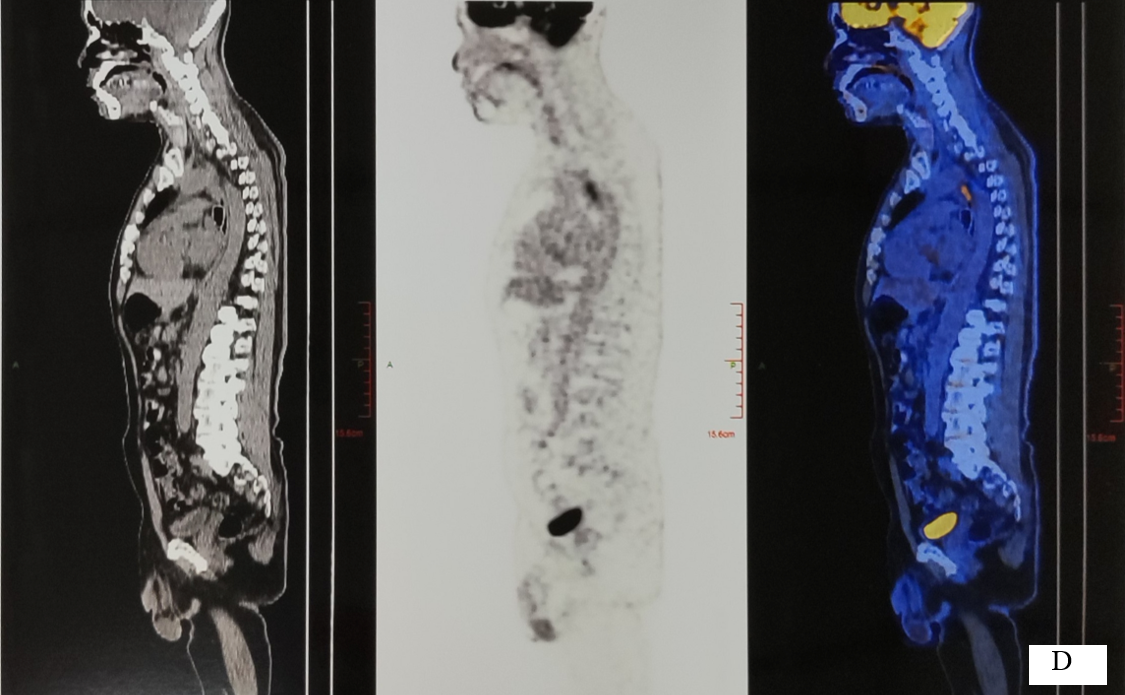


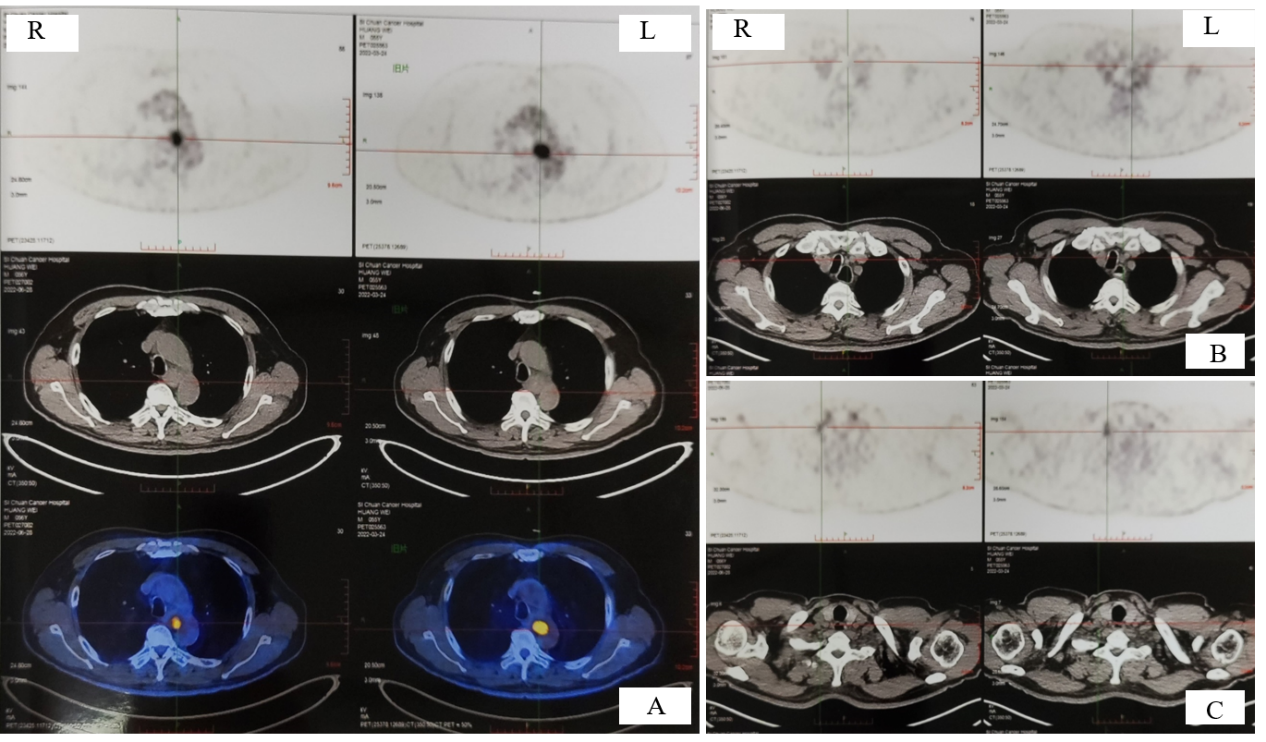
Figure 1: PET-CT Imaging Representation. (A) (A) The metabolic imaging comparison of the primary tumor site, after four courses of treatment (right image) against the initial diagnosis, shows a reduced metabolic activity with the maximum SUV dropping from 11.3 initially to 9.4 following the treatment. (B) The post-therapy metabolic imaging of the upper left tracheal lymph node (right image), when compared to the initial diagnosis (left image), reveals a modest decrease in size and metabolic rate with the maximum SUV decreasing from 2.6 to 2.4 post-treatment. (C) The comparative metabolic image of the right supraclavicular lymph node post-four treatment cycles (right side) against the baseline (left side) demonstrates a slight reduction in size with the metabolic rate unchanged, keeping the maximum SUV constant at 2.8. (D) Sagittal view image. Annotation: At initial diagnosis of the abdominal and pelvic areas: An observation of a low-density lesion measuring roughly 9mm by 6mm in the right posterior lobe of the liver, with no aberrant uptake detected. The rest of the hepatic parenchyma exhibited a maximum SUV of 3.8 and an average SUV of 2.7, with no unusual uptakes noted in the remaining abdominal and pelvic tissues. Following four cycles of therapy, in the abdominal and pelvic area: The imaging showed a persistent low-density lesion about 9mm*6mm in size in the right posterior lobe of the liver, no abnormal uptake was detected, and the remaining liver tissue showed a reduction in maximum SUV to 3.3 and average SUV to 2.3, with no atypical uptakes in the rest of the abdominopelvic region.
